# Bioinspired laminated bioceramics with high toughness for bone tissue engineering

**DOI:** 10.1093/rb/rbac055

**Published:** 2022-08-22

**Authors:** Jinzhou Huang, Dong Zhai, Jianmin Xue, Tian Li, Dudi Ren, Chengtie Wu

**Affiliations:** State Key Laboratory of High Performance Ceramics and Superfine Microstructure, Shanghai Institute of Ceramics, Chinese Academy of Sciences, Shanghai 200050, P.R. China; Center of Materials Science and Optoelectronics Engineering, University of Chinese Academy of Sciences, Beijing 100049, P.R. China; State Key Laboratory of High Performance Ceramics and Superfine Microstructure, Shanghai Institute of Ceramics, Chinese Academy of Sciences, Shanghai 200050, P.R. China; State Key Laboratory of High Performance Ceramics and Superfine Microstructure, Shanghai Institute of Ceramics, Chinese Academy of Sciences, Shanghai 200050, P.R. China; State Key Laboratory of High Performance Ceramics and Superfine Microstructure, Shanghai Institute of Ceramics, Chinese Academy of Sciences, Shanghai 200050, P.R. China; State Key Laboratory of High Performance Ceramics and Superfine Microstructure, Shanghai Institute of Ceramics, Chinese Academy of Sciences, Shanghai 200050, P.R. China; State Key Laboratory of High Performance Ceramics and Superfine Microstructure, Shanghai Institute of Ceramics, Chinese Academy of Sciences, Shanghai 200050, P.R. China; Center of Materials Science and Optoelectronics Engineering, University of Chinese Academy of Sciences, Beijing 100049, P.R. China

**Keywords:** laminated bioceramics, MXene, bioinspired structure, high toughness, bone tissue engineering

## Abstract

For the research of biomaterials in bone tissue engineering, it is still a challenge to fabricate bioceramics that overcome brittleness while maintaining the great biological performance. Here, inspired by the toughness of natural materials with hierarchical laminated structure, we presented a directional assembly-sintering approach to fabricate laminated MXene/calcium silicate-based (L-M/CS) bioceramics. Benefiting from the orderly laminated structure, the L-M/CS bioceramics exhibited significantly enhanced toughness (2.23 MPa·m^1/2^) and high flexural strength (145 MPa), which were close to the mechanical properties of cortical bone. Furthermore, the L-M/CS bioceramics possessed more suitable degradability than traditional CaSiO_3_ bioceramics due to the newly formed CaTiSiO_5_ after sintering. Moreover, the L-M/CS bioceramics showed good biocompatibility and could stimulate the expression of osteogenesis-related genes. The mechanism of promoting osteogenic differentiation had been shown to be related to the Wnt signaling pathway. This work not only fabricated calcium silicate-based bioceramics with excellent mechanical and biological properties for bone tissue engineering but also provided a strategy for the combination of bionics and bioceramics.

## Graphical Abstract

**Figure:**
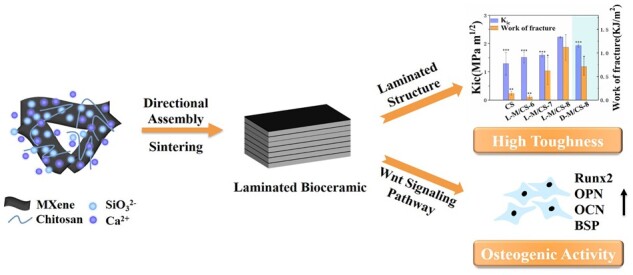

## Introduction

Bone tissue is the shield for protecting internal organs while also plays an important role in homeostasis of the body. When suffering from large bone defects, it is difficult to achieve completely healing by bone tissues itself and requires external surgical intervention [1]. Although the bone autografts or allografts are the gold standard treatment for repairing bone defects, the limited donor resources or immune-related complications have not been addressed in clinical [2]. Since the introduction of bone tissue engineering [3], great efforts have been made to design artificial biomaterials for replacement of allogeneic bone and xenogeneic bone [1, 4, 5]. Among these artificial biomaterials, bioceramics have gained much attention due to easy preparation, excellent degradability and remarkable osteogenic bioactivity [6, 7]. In particular, silicate bioceramics, as a novel bioceramics system, can significantly stimulate the osteogenic and angiogenic differentiation of cells and promote the regeneration of bone tissues by the release of Si-containing ionic products [8–11]. However, different from the high toughness of bone, the intrinsic brittleness of bioceramics leads to the mismatch of toughness between silicate bioceramics and bone tissue, which has greatly limited the clinical application of silicate bioceramics.

To improve the toughness of ceramics, many basic approaches have been proposed and achieved favorable effect, such as reinforced by fibers or whiskers, microcrack induced toughening or phase transformation toughening [12–15]. Besides these traditional toughening methods, the toughening effect of hierarchical structures of natural materials has also attracted much attention in recent years [16–18]. Many studies have demonstrated that the outstanding toughness of natural materials is closely related with orderly laminated structure from nanoscale to macroscale [19–22]. For example, nacre, one of the ultra-tough and ultra-strong natural materials, possesses the laminated structure of alternating layered aragonite platelets and organic materials. Besides, Haversian system, the basic unit of cortical bone, also consists of concentric layered structure. Such laminated structures have been considered as the primary reason for the toughness improvement to these natural materials [23, 24]. Once the fracture has begun, the plastic deformation at crack tips, crack deflection and other mechanical behaviors on the laminated structures can dissipate energy and thus improve strength and toughness of materials [25]. At the microscale level, the crack redirection and twisting occur at the weak interface in laminated structure when the ratio of modulus of the two components is larger than five according to some research [20]. Moreover, in some laminated structure, the organic phase allows strain redistribution, and thus improves toughness [26]. Therefore, it is a potential strategy for improving the toughness of silicate bioceramics by constructing biomimetic laminated structure.

There are several strategies to construct the laminated structure, such as directional assembly, layer-by-layer assembly, freeze casting and assembly mineralization [27–30]. Among them, the directional assembly based on 2D templates is one of the most simple and effective ways to fabricate laminated bulk materials. Two-dimensional nanosheets, such as Graphene, MXene or MoS_2_, could be regarded as the directional templates for the mineralization and deposition of inorganics.

**Figure 1.**
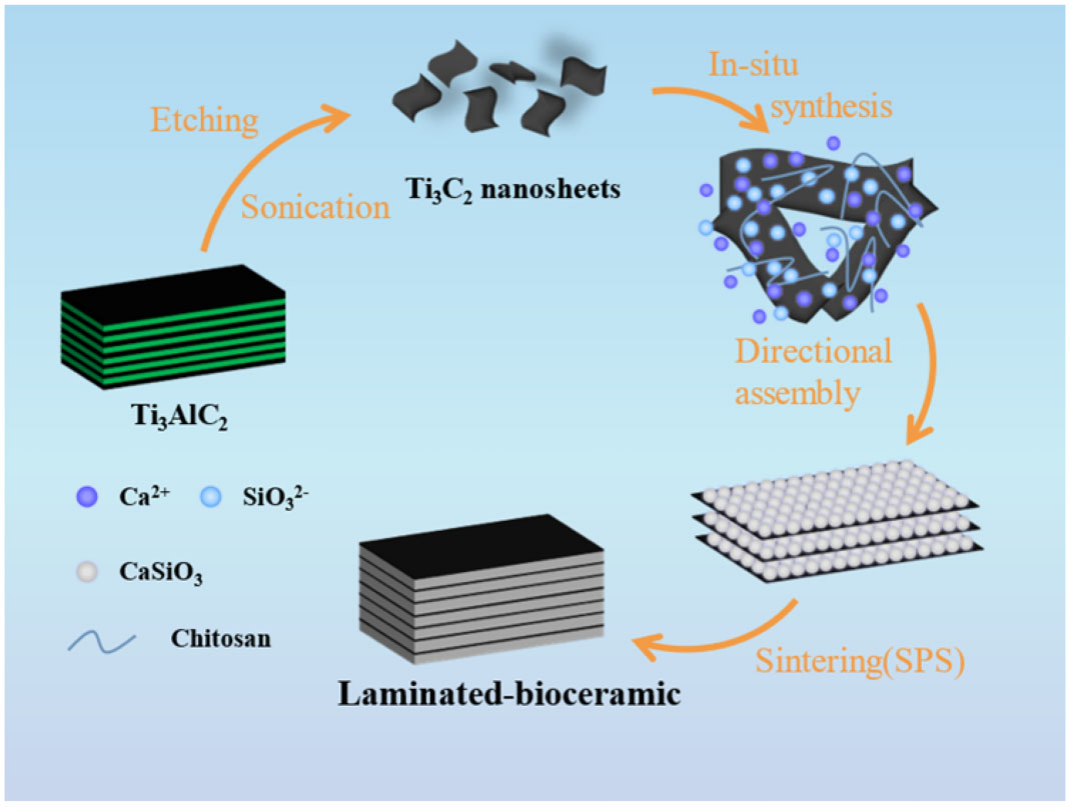
Schematic illustration of the synthesis of bioinspired laminated bioceramics. The L-M/CS bioceramics were fabricated through directional assembly-sintering approach based on MXene templates.

Herein, inspired by the toughening of laminated structure of natural materials, we presented a directional assembly-sintering approach based on MXene nanosheets to prepare laminated MXene/calcium silicate-based (L-M/CS) bioceramics (Fig. 1). The L-M/CS bioceramics showed superior toughness and flexural strength due to the toughening effects of laminated structure by redirecting crack propagation and promoting crack branch. Moreover, the newly formed phase of L-M/CS bioceramics during sintering improved the chemical stability and avoided the rapid degradation of CaSiO_3_. Furthermore, the prepared L-M/CS bioceramics could significantly promote proliferation and adhesion of rabbit bone marrow mesenchymal stem cells (rBMSCs) and stimulate the expression of osteogenesis-related genes, which proved that the laminated bioceramics had an excellent biocompatibility. Such high-performance bioinspired laminated bioceramics were believed to be potential candidates for bone tissue engineering.

## Materials and methods

### Materials

Ti_3_AlC_2_ powders were purchased from 11 Technology Co., Ltd (China). Chitosan, sodium metasilicate nonahydrate (Na_2_SiO_3_·9H_2_O), hydrochloric acid and ammonia solution were purchased from Sinopharm Chemical Reagent Co., Ltd (China). Calcium nitrate tetrahydrate [Ca(NO_3_)_2_·4H_2_O] and lithium fluoride (LiF) were purchased from Shanghai Aladdin Biochemical Technology Co., Ltd (China).

### Preparation of MXene

The MXene was prepared by etching Ti_3_AlC_2_ powders. The Ti_3_AlC_2_ powders were immersed and stirred in HCl (9 M) and LiF (5 g/100 ml) solution for 24 h at room temperature. Then, the mixed solution was centrifuged at 4500 rpm and washed with deionized water until the pH value reached 6.0. The final precipitation was immersed in ethanol and sonicated with an ultrasound machine for an hour. Then, the solution was centrifuged at 11 000 rpm for 10 min to obtain the precipitation. The precipitation was dispersed in deionized water and sonicated for 20 min to dissolve MXene in water. Finally, after centrifuging at 4000 rpm for 3 min, the MXene solution was obtained and saved at 4°C.

### Preparation of L-M/CS bioceramics

Chitosan solution (5 mg·ml^−1^) was added into MXene solution and stirred for 1 h. Then, the Ca(NO_3_)_2_·4H_2_O was mixed with the above solution and sonicated for 1 h to get the mixed solution without suspended solids. After stirring for 2 h, Na_2_SiO_3_·9H_2_O solution was added slowly through separatory funnel. Then, the pH value was adjusted to 13 by ammonia solution. After stirring for 12 h at room temperature, the mixed solution was filtered to form green body. The shape of green body was cylinder with diameter of 6 cm. There was no washing process before filtration for avoiding damaging the orderly structure. After drying at 60°C, the green body was sintered at 950°C and 40 MPa by the spark plasma sintering (SPS) technique (SPS-725, Japan). Samples with different contents could be synthesized by changing the proportion of Ca(NO_3_)_2_·4H_2_O and Na_2_SiO_3_·9H_2_O.

### Preparation of D-M/CS bioceramics

The process for preparing D-M/CS bioceramics was similar to the L-M/CS bioceramics before filtration. After drying, the green body of D-M/CS was ground into powder and prepressed in cylindrical mold (diameter: 20 mm) at 20 MPa. Then the sample was sintered at the same conditions with L-M/CS bioceramics.

### Preparation of CS bioceramics

Chitosan solution was immersed and stirred in deionized water for 1 h. Then, Ca(NO_3_)_2_·4H_2_O was dissolved in the solution. After stirring for 2 h, Na_2_SiO_3_·4H_2_O solution was added slowly through separatory funnel. The ammonia solution was used to adjust pH value to 13. The mixed solution, after stirring for 12 h at room temperature, was vacuum filtered to obtain green body. The green body was sintered at the same condition with the L-M/CS bioceramics after drying at 60°C.

### Characterization of samples

The thickness of MXene nanosheet was evaluated by the atomic force microscopy (AFM, NTEGRA, Russia). The microstructures of the bioceramics were observed by the scanning electron microscopy (SEM, S-4800, SU8220, Japan). The microstructure of MXene nanosheet was obtained through transmission electron microscopy (TEM, Tecnai G2 F20, Netherlands). X-ray diffraction (XRD, D8 ADVANCE, Germany) and TG-DSC (Thermogravimetric-Differential scanning calorimeter) were used to analyze the component of samples.

### Mechanical testing

The mechanical properties were measured by standard mechanical testing machine (Instron-5566, USA). Through three-point test, the flexural strength could be obtained. Moreover, the modulus and work of fracture could be calculated through stress-strain curve. The samples were cut into specimens (3 × 3 × 15 mm) for testing. Then specimens were performed at a loading speed of 0.5 mm·min^−1^ and a span of 10 mm. The flexural strength and modulus could be obtained from the highest point in the stress-strain curve and the slope of the curve, respectively. The work of fracture could be calculated by determining the area under the load-displacement curve and dividing by the area of the fracture surface. The specimens were prenotched about 0.75 mm for SNEB (single edge notched beam specimen techniques) test with a loading speed of 0.05 mm·min^−1^ and a span of 10 mm. Fracture toughness (*K_ic_*) was calculated by the equation:

$$\mathrm{Kic}=\frac{3\times 0.0316P\mathrm{icS}\sqrt{\frac{a}{W}}\left[1.99-\frac{a}{W}\left(1-\frac{a}{W}\right)\left(2.15-3.93\frac{a}{W}+2.7{\left(\frac{a}{W}\right)}^{2}\right)\right]}{2BW^{\frac{3}{2}}(1+\frac{2a}{W}){\left(1-\frac{a}{W}\right)}^{\frac{3}{2}}}.$$

*P_ic_*, *S*, *a*, *W* and *B* stand for the maximum load, support span, notch depth, thickness and width, respectively. The number of samples in each group was 5.

### Degradation experiments

The L-M/CS bioceramics were soaked in Tris-HCl solutions (pH = 7.4) to explore their degradability, including the ions release, the change of pH value and weight loss. To investigate the ions release, the bioceramics were soaked into Tris-HCl for 1, 4, 7 and 14 days with the ratio of the solution volume to the bioceramics mass was 100 ml·g^−1^. The solution was put in shaker with the temperature of 37°C and the shaking frequency of 100 rpm. The concentrations of the release ions were measured by inductive coupled plasma atomic emission spectrometry (ICP, Varian 715-ES, USA). The quality of samples was weighed on Days 7 and 14. Moreover, the pH value of the soaked Tris-HCl was measured by pH meter at specific time points. The number of samples in each group was 7.

### Cell culture

The rBMSCs were provided by Cyagen Biosciences. The rBMSCs were cultured in Dulbecco’s modified Eagle’s medium low glucose (Thermo Fisher Scientific, Grand Island, USA) supplemented with 10% fetal bovine serum (FBS; Thermo Fisher Scientific) and 1% (v/v) penicillin/streptomycin (Thermo Fisher Scientific) at 37°C in a humidified CO_2_ incubator. The medium was changed every day. The cells were passaged upon reaching around 90% confluence and expanded through two passages before being used in the following experiments.

### Cell attachments

The rBMSCs were used in this study. To observe the morphology of rBMSCs on different materials, cells were seeded at a density of 1 × 10^4^ cells per well on the L-M/CS-8 and CS in 24-well culture plates and cultured for 3 days. The samples were processed with 2.5% glutaraldehyde, ethanol solution (30, 50, 70, 80, 90, 95 and 100 v/v %), 1:1 mixed solution (ethanol: HMDS (Hexamethyl disilylamine) v/v) and HMDS. SEM was used to observe cell morphology. To better observe the cell adhesion, rBMSCs were seeded at a density of 1 × 10^4^ cells per well on the L-M/CS-8 and CS in 24-well culture plates and cultured for 3 days. The cellular samples were proceeded with 4% paraformaldehyde for 20 min. FITC (Fluorescein isothiocyanate)-conjugated phalloidin (Molecular Probes, USA) was used for labeling the cytoskeleton for 20 min at room temperature. Cellular specimens were washed thrice in PBS (Phosphate buffer saline) and treated with 1 μg·ml^−1^ of DAPI in PBS for 5 min to stain the cell nucleus. Subsequently, the confocal images were obtained by confocal laser scanning microscopy (CLSM, TCS SP8, Leica, Germany) to observe cell adhesion.

### Cell proliferation

To evaluate cell proliferation of the L-M/CS bioceramics, rBMSCs were seeded at a density of 1 × 10^4^ cells per well on the L-M/CS-8 and CS in 24-well culture plates and cultured for 1, 4 and 7 days. Cells were incubated with 10% CCK-8 solution (Dojindo, Japan) for 2 h at 37°C in 5% CO_2_ incubator according to the manufacturer’s instructions. Subsequently, the absorbance of medium was determined by a microplate reader (Spark, Tecan, Switzerland) at 450 nm. The number of samples in each group was 5.

### Osteogenesis-related and Wnt signaling pathway-related gene expression

To investigate the effect of L-M/CS-8 on the expression of osteogenesis-related and Wnt signaling pathway-related genes of rBMSCs, rBMSCs were seeded at a density of 1 × 10^4^ cells per well on L-M/CS-8 and CS in 24-well culture plates and cultured for 7 days. The rBMSCs were treated with Trizol Reagent (Invitrogen Pty Ltd, Australia) according to the manufacturer’s instructions. The total RNA was extracted using chloroform extraction and isopropanol precipitation. The RNA concentration was measured at 260 nm by NanoDrop 2000 reader (Thermo Fisher Scientific, USA). Then cDNA was obtained by the RNA reverse transcription using First strand cDNA Synthesis kit (TOYOBO, Japan). Real-time PCR analysis was performed on StepOnePlus Real-Time PCR Systems (Applied Biosystems, USA) using SYBR Green QPCR Master Mix (TaKaRa, Japan). The cycling conditions were 30 s of polymerase activation at 95°C followed by 40 cycles at 95°C for 5 s and 60°C for 30 s. The housekeeping gene GAPDH was used to normalize the results. The quantitative analysis of relative genes was measured by a 2^−ΔΔCT^ method. Osteogenesis-related genes were Runx2, OPN, OCN and BSP and β-catenin, CK1, APC, Axin and GSK-3β were used as indicators of activation of Wnt signaling pathway. To examine the effect of the Wnt signaling pathway in L-M/CS-8-stimulated osteogenesis-related gene expression, cells were treated with the Wnt signaling pathway inhibitor Dickkopf-1 (DKK1, PeproTech, USA) in 0.5 μg·ml^−1^ for 7 days. Real-time PCR analysis was performed by the same methods as described above. The sequences of primers for the above genes were shown in Supplementary Table S1. The number of samples in each group was 4.

### ALP staining and ALP activity assay

The rBMSCs were seeded at a density of 1 × 10^4^ cells per well in 12-well culture plates. The transwell inserts (0.4 μm pore size, Millipore) were placed on each well. Sterile L-M/CS-8 and CS bioceramics were added to the top portion of a transwell insert. For ALP (Alkaline phosphatase) staining, cells were cultured for 3 days and then fixed with 4% paraformaldehyde for 20 min. Cells were washed twice with PBS and stained using the BCIP/NBT Alkaline Phosphatase Color Development Kit (Beyotime, Shanghai, China) according to the manufacturer’s instructions. For measurement of ALP activity, cells were cultured for 3 days and lysed with lysis buffer consisting of 20 mM Tris-HCl (pH = 7.5), 150 mM NaCl, and 1% Triton X-100. ALP activity was determined using the Alkaline Phosphatase Assay Kit (Beyotime, Shanghai, China) according to the manufacturer’s instructions. Briefly, the conversion of colorless p-nitrophenyl phosphate to colored p-nitrophenol was measured at 405 nm with a microplate reader (Spark, Tecan, Switzerland). The total protein content was determined using the BCA protein assay kit (Pierce Biotechnology, USA). ALP activity was calculated as the OD (Optical density) value divided by the reaction time and the total protein content. To examine the effect of the Wnt signaling pathway in L-M/CS-8-stimulated ALP activity, cells were treated with the Wnt signaling pathway inhibitor Dickkopf-1 (DKK1, PeproTech, USA) in 0.5 μg·ml^−1^ for 3 days. ALP activity was assayed by the same methods as described above. The number of samples in each group was 3.

### Immunofluorescence images of rBMSCs

To investigate the expression of Wnt signaling pathway-related protein in rBMSCs, a confocal laser scanning microscope was used to acquire immunofluorescence images. Briefly, rBMSCs were seeded at a density of 1 × 10^4^ cells per well on the L-M/CS-8 and CS in 24-well culture plates and cultured for 3 days. Four percent paraformaldehyde was used to fix rBMSCs. The rBMSCs were permeabilized with 0.1% Triton X-100 and blocked with 1% BSA (Bovine albumin) solution at 37°C for 1 h. Subsequently, cellular specimens were incubated with β-catenin primary antibody (Santa Cruz Biotechnology, USA) at 4°C overnight. After washing thrice in PBS buffer, the cells were incubated with Alexa Fluor 647-conjugated goat-anti-rabbit IgG (Cell Signaling Technology, USA) for 1 h at room temperature. FITC-conjugated phalloidin (Molecular Probes, USA) was used for labeling the cytoskeleton for 20 min at room temperature. Cellular specimens were washed thrice in PBS and treated with 1 μg·ml^−1^ of DAPI in PBS for 5 min to stain the cell nucleus. Immunofluorescence images were obtained using confocal microscope. To examine the effect of the Wnt signaling pathway in L-M/CS-8-stimulated β-catenin expression, cells were treated with the Wnt signaling pathway inhibitor Dickkopf-1 (DKK1, PeproTech, USA) in 0.5 μg·ml^−1^ for 3 days. Immunofluorescence staining was performed by the same methods as described above.

### Statistics analysis

All of the data were showed as mean ± standard deviation (SD). A Student’s *t*-test analysis of variance was used to determine the statistical significance. A *P*-value < 0.05 was considered significance difference. The data were indicated with (*) for *P *<* *0.05, (**) *P *<* *0.01, (***) *P *<* *0.001.

## Results and discussion

### Fabrication strategy and structural characterization

**Figure 2.**
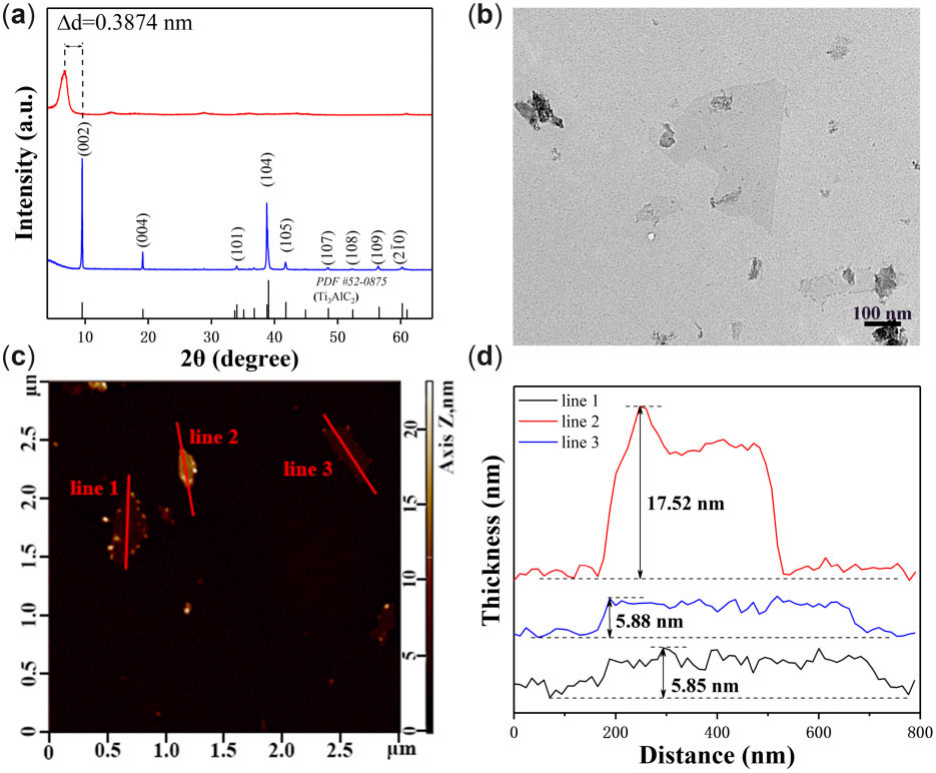
(**a**) XRD patterns of Ti_3_AlC_2_ powder and Ti_3_C_2_ nanosheets. (**b**) TEM image and (**c**) AFM image of Ti_3_C_2_ nanosheets after etching. (**d**) AFM-measured thickness of nanosheets. The MXene nanosheets were successfully prepared by etching MAX ceramics.

MXene, a kind of ultrathin nanomaterials derived from MAX ceramics (Ti_3_AlC_2_, Ta_4_AlC_3_, Nb_2_AlC, etc.) [31], is widely used on biological and biomedical application as an inorganic nano-systems due to its remarkable biological properties [32–34]. In this work, 2D MXene (Ti_3_C_2_) nanosheets were prepared by etching MAX phase according to the reported method [35]. The XRD pattern of MAX phase and Ti_3_C_2_ nanosheets revealed that the peak intensities originating from MAX phase decreased substantially after etching (Fig. 2a). Especially, the (002) peak broadened and downshifted to a lower angle, which was typical representation of etched MXene and implied the conversion of MAX ceramic to MXene [32]. TEM images showed that the thin and electron-transparent Ti_3_C_2_ nanosheets were successfully prepared (Fig. 2b). The average thickness of Ti_3_C_2_ nanosheets was about 6 nm (Fig. 2c and d).

**Figure 3.**
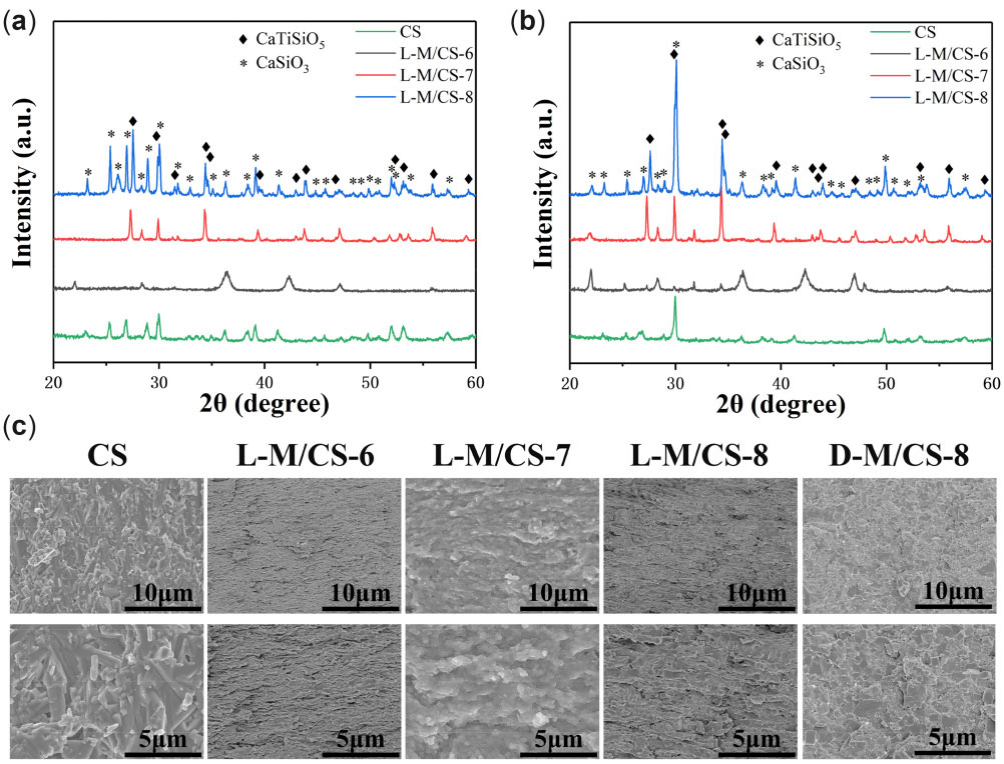
The composition and microstructure of prepared bioceramics. XRD pattern of (**a**) surface and (**b**) section of laminated bioceramics. (**c**) SEM images of cross-sectional microstructure of laminated bioceramics in different magnification. The prepared L-M/CS-6 and L-M/CS-8 bioceramics exhibited the laminated microstructure.

The MXene nanosheets were further used as 2D templates and mixed with *in situ* synthesized calcium silicates in solution. Subsequently, the mixed solution was filtrated to form ceramic green body under the induction of MXene templates during directional assembly process. Finally, the ceramic green body was sintered to fabricate L-M/CS bioceramic by SPS. The L-M/CS bioceramics with calcium silicate mass ratios of 60%, 70%, 80% and 100% were named as L-M/CS-6, L-M/CS-7, L-M/CS-8 and CS, respectively. XRD patterns and TG-DSC curve were used to determine the phase composition of sintered samples (Supplementary Fig. S1). It was found that the phase of CaTiSiO_5_ was formed after sintering for L-M/CS-7 and L-M/CS-8 bioceramics, which might be due to the reaction of CaSiO_3_ and Ti_2_C_3_ nanosheets under high-temperature sintering (Fig. 3a and b). The newly formed phase might affect the chemical stability and biological properties of calcium silicate bioceramics, which would be discussed in later. Subsequently, the cross-section morphology and surface microstructure of L-M/CS bioceramics were further characterized to confirm the formation of laminated microstructure (Fig. 3c and Supplementary Fig. S2). It was found that L-M/CS-8 and L-M/CS-6 bioceramics had laminated structure at the micron scale (Supplementary Fig. S3). The laminated structure in L-M/CS-7 bioceramics was not clearly observed as compared with other two L-M/CS bioceramics (Supplementary Fig. S4). On the contrary, the CS bioceramics without MXene do not have orderly microstructure. In addition, it was observed that the L-M/CS bioceramics had the smaller grain in compared with the CS bioceramics. As a comparison, the D-M/CS-8 bioceramics, which had the same component but different structure with L-M/CS-8 bioceramics, were also fabricated by the similar method with L-M/CS-8 except that the green body was ground into powder after directional assemble process. Despite the presence of MXene, the D-M/CS-8 bioceramics also did not form laminated microstructure. Therefore, both 2D MXene templates and directional assemble process played pivotal roles in the formation of laminated microstructure. Above all, the L-M/CS bioceramics with laminated microstructure were successfully prepared by the directional assembly-sintering method.

### Mechanical properties

**Figure 4.**
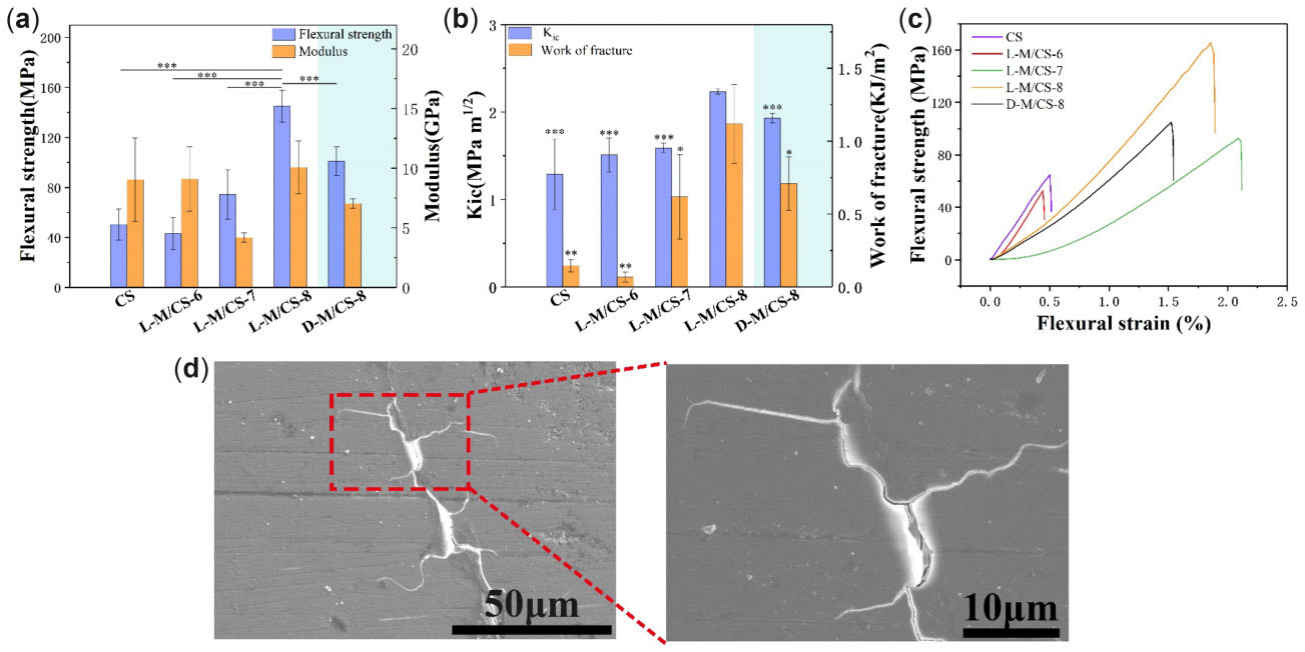
Characterization of mechanical properties of laminated bioceramics. (**a**) Flexural strength and modulus of CS, D-M/CS and different L-M/CS bioceramics (*n* = 5). (**b**) Comparison of fracture toughness and work of fracture for the CS, D-M/CS and different L-M/CS bioceramics (*n* = 5). (**c**) Flexural stress-strain curves of different bioceramics. (**d**) The crack deflection and crack branch during fraction of L-M/CS-8 bioceramics. The results showed that the L-M/CS-8 had the better mechanical properties than traditional CaSiO_3_ bioceramics and exhibited similar modulus and strength with cortical bone, which indicated the laminated bioceramics were to be potential candidates for bone implants. (*P < 0.05, **P < 0.01, ***P < 0.001)

Although calcium silicates had received great attention due to the tissue regeneration, the brittleness prevented their further application on load-bearing bone regeneration. The prepared L-M/CS bioceramics showed significantly enhanced toughness and strength and proper modulus. As shown in Fig. 4a and b, it was found that the mechanical properties of L-M/CS bioceramics increased with the decreased of the mass ratio of MXene. The flexural strength of CS, L-M/CS-6 and L-M/CS-7 were lower than 75 MPa. On the contrary, the flexural strength of L-M/CS-8 reached 145 MPa, which was about 2.9 times higher than CS and match with the cortical bone (50–150 MPa) [36]. The modulus of ideal bone implants should match the modulus of cortical bone to avoid bone resorption and implant failure induced by stress shielding effects [37]. The modulus of L-M/CS-8 bioceramics was about 10.07 GPa and matched that of cortical bone (7–30 GPa) [36], which was important to avoid the stress shielding after implanting. More importantly, the toughness of L-M/CS-8 had gained significantly improvement and reached 2.23 MPa·m^1/2^, which was much higher than that of L-M/CS-6 (1.51 MPa·m^1/2^), L-M/CS-7 (1.59 MPa·m^1/2^) and CS (1.29 MPa·m^1/2^). The fracture work of L-M/CS-8 bioceramics reached 1119 J·m^−2^, which was about 7.7 times higher than CS bioceramics (145 J·m^−2^). Moreover, the flexural strength and toughness of L-M/CS-8 bioceramics increased almost by 43% and 16% in compared with D-M/CS-8, indicating that the laminated structure had a valuable influence on the mechanical properties of L-M/CS bioceramics.

To explore the toughening mechanism of L-M/CS-8, we further conducted the fracture analysis. As described before, the laminated structure of materials had a certain influence on the crack extension (such as crack deflection and crack branch) during the process of fracture, which could significantly promote the toughness of materials. Similarly, Fig. 4d showed that the crack deflections and crack branches occurred during the fracture of L-M/CS-8 bioceramics, which was favorable to energy dissipation and thus promoted toughness of prepared bioceramics. Overall, the L-M/CS bioceramics possessed excellent toughness and strength similar to cortical bone, successfully avoiding the brittleness of calcium silicate bioceramics.

### Degradation performance

As ideal bone tissue engineering materials, the degradation rate of materials should be compatible with the rate of bone growth. However, traditional calcium silicate bioceramics had rapid degradation rate and easily led to the rapid increase of pH value in regional microenvironment, which was not conducive to bone regeneration [38, 39]. As described before, there was new phase of CaTiSiO_5_ in L-M/CS bioceramics after sintering, which might cause the changes in the degradability of L-M/CS bioceramics.

**Figure 5.**
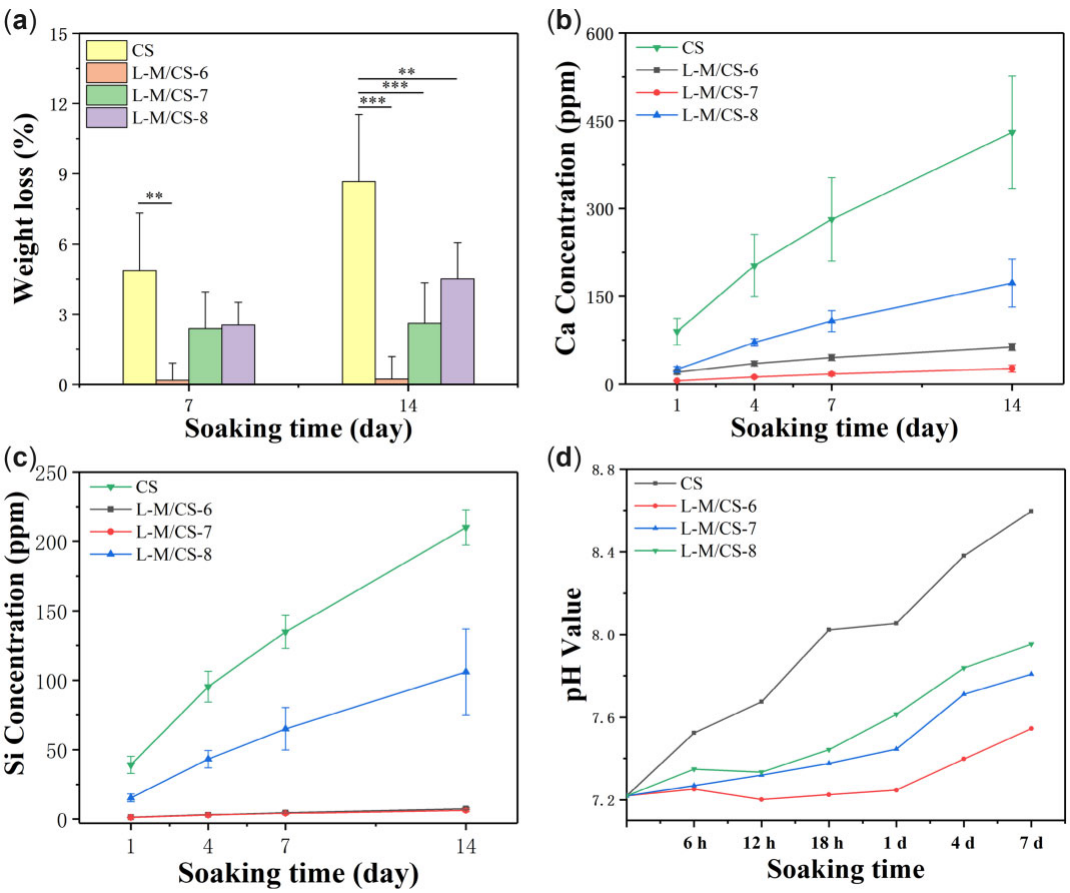
The degradability of bioceramics. The change of (**a**) weight loss, (**b, c**) ions concentration and (**d**) pH value of samples after soaking with Tris-HCl (*n* = 7). The L-M/CS bioceramics had better chemical stability than CS and could release Ca and Si ions. (*P < 0.05, **P < 0.01, ***P < 0.001)

To investigate the degradability of L-M/CS bioceramics, the prepared samples were soaked in Tris-HCl for various time points. As shown in Fig. 5a, the weight loss of three different L-M/CS bioceramics was significantly lower than that of pure CS bioceramics. After soaking for 14 days, the weight loss of CS bioceramics reached about 8.6%. On the contrary, the weight loss of L-M/CS-7 and L-M/CS-8 was about 2.6% and 4.5%, respectively. However, L-M/CS-6 bioceramics were too stable to degrade, which had weight loss of only 0.23%. The concentration of Ca and Si ions released from bioceramics were determined by inductive coupled plasma emission spectrometer (ICP) analysis. Similar to the condition of weight loss, the Ca and Si ions concentrations of CS bioceramics were much higher than that of L-M/CS bioceramics. The Ca and Si ions concentration of CS reached about 430 and 210 ppm, respectively, while the Ca and Si ions concentration of L-M/CS-8 was about 173 and 106 ppm after soaking for 14 days (Fig. 5b and c). The slow degradation rate of L-M/CS bioceramics also avoided the rapid increase of pH value in soaked solution. The pH value of L-M/CS-8 group kept blow 8.0, while the pH value of CS group reached about 8.6 after soaking for 14 days (Fig. 5d). It was reported that CaTiSiO_5_ exhibited better chemical stability in compared with CaSiO_3_ [40, 41]. Therefore, the promotion of stability of prepared L-M/CS-8 bioceramic could be attributed to the formation of new phase (CaTiSiO_5_) during sintering. Above all, the L-M/CS-8 bioceramics possessed more suitable degradability than traditional CaSiO_3_ bioceramics. Considering the mechanical and degradation performance, the L-M/CS-8 bioceramics were selected to further investigate *in vitro* biological properties.

### *In vitro* biological properties

**Figure 6.**
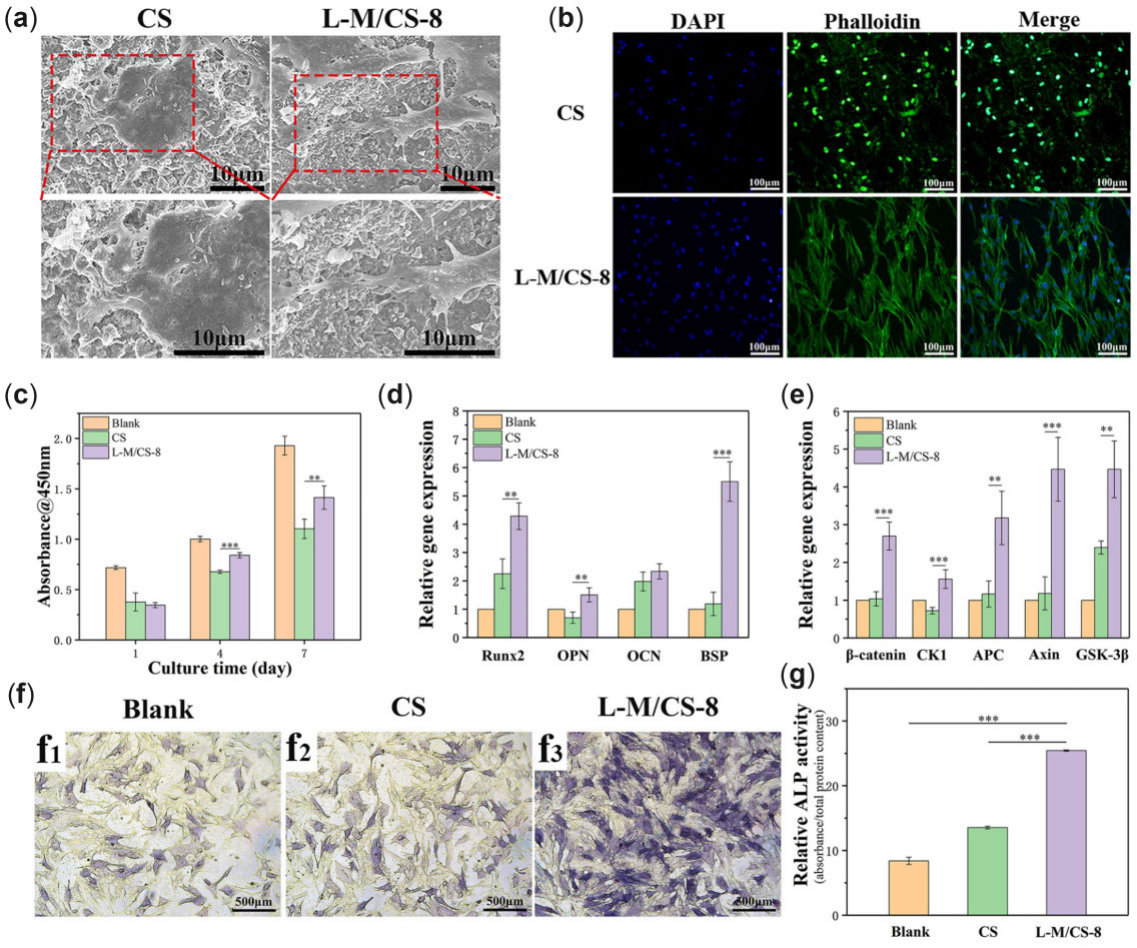
Adhesion, proliferation and differentiation of rBMSCs cultured with L-M/CS-8 and CS. (**a**) SEM images and (**b**) confocal images of the cells cultured with L-M/CS-8 and CS for 3 days. (**c**) Proliferation of rBMSCs cultured with different materials (*n* = 5). (**d**) Osteogenesis-related genes and (**e**) Wnt signaling pathway-related genes expression of cells cultured with different materials for 7 days (*n* = 4). (**f**) ALP staining and (**g**) ALP activity of cells cultured for 3 days (*n* = 3). The L-M/CS-8 displayed better effect on promotion of adhesion, proliferation and the expression of osteogenesis-related genes for rBMSCs than CS bioceramics. (*P < 0.05, **P < 0.01, ***P < 0.001)

The cell attachment, cell proliferation, gene expression and ALP activity assays were conducted to determine the *in vitro* biological properties of L-M/CS-8 bioceramics. The rBMSCs were cultured with L-M/CS-8 and CS. Figure 6a showed the morphology of rBMSCs after cultured with L-M/CS-8 or CS for 3 days. It could be observed the cells cultured with L-M/CS-8 bioceramics spread well and adhered to the surface of bioceramics. In addition, compared with cells cultured with CS, cells cultured with L-M/CS-8 exhibited better morphology with more pseudopodia. The confocal microscopy images showed the cells cultured with L-M/CS-8 revealed well-spread morphologies (Fig. 6b). On the contrary, cells on the surface of CS exhibited poor spherical state. The morphology of cells reflected cells survival state, which the cells with poor state might have a significant influence on cell proliferation and differentiation. For cell proliferation, both L-M/CS-8 and CS promoted cell proliferation with the increase of culture time. Moreover, L-M/CS-8 revealed better cell viability than CS, which might be due to the stable environment bring by suitable degradability of L-M/CS-8 (Fig. 6c).

Further research found that L-M/CS-8 had a promoting effect on the expression of osteogenesis-related genes, including Runx2, OPN, OCN and BSP, which indicated that L-M/CS-8 could significantly enhance osteogenic differentiation of rBMSCs in compared with CS (Fig. 6d). ALP activity was known as an early marker for osteoblastic differentiation [42, 43]. As shown in Fig. 6f and g, ALP activity of cells cultured with L-M/CS-8 was improved significantly than cultured with CS, which proved that L-M/CS-8 could enhance early osteogenic differentiation of rBMSCs. It was reported that Ca, Si and other bioactive ions can enhance metabolic activity of cells, but the excess ions concentration might have opposite effect [44–47]. Therefore, the Si and Ca ions released from L-M/CS-8 bioceramics might facilitate the promotion of osteogenesis activity.

Several researches had proved that Wnt signaling pathway exhibited significant effect on the bone development, homeostasis and regulation of bone mineral density [48–50]. The pathway played an important role in the osteogenic differentiation of mesenchymal stem cells [51]. The induction of Wnt signaling pathway enhanced the formation of bone while the inactivation of the pathway resulted osteopenia [52, 53]. To further explore the mechanisms that promoted osteogenic differentiation of L-M/CS-8, the expression of Wnt signaling pathway-related genes was evaluated. Figure 6e showed L-M/CS-8 could obviously promote the expression of Wnt signaling pathway including β-catenin, CK1, APC, Axin and GSK-3β. It was reasonable to speculate that the enhancement to osteogenesis-related genes expression of L-M/CS-8 could be attributed to promotion to the expression of Wnt signaling pathway-related genes.

**Figure 7.**
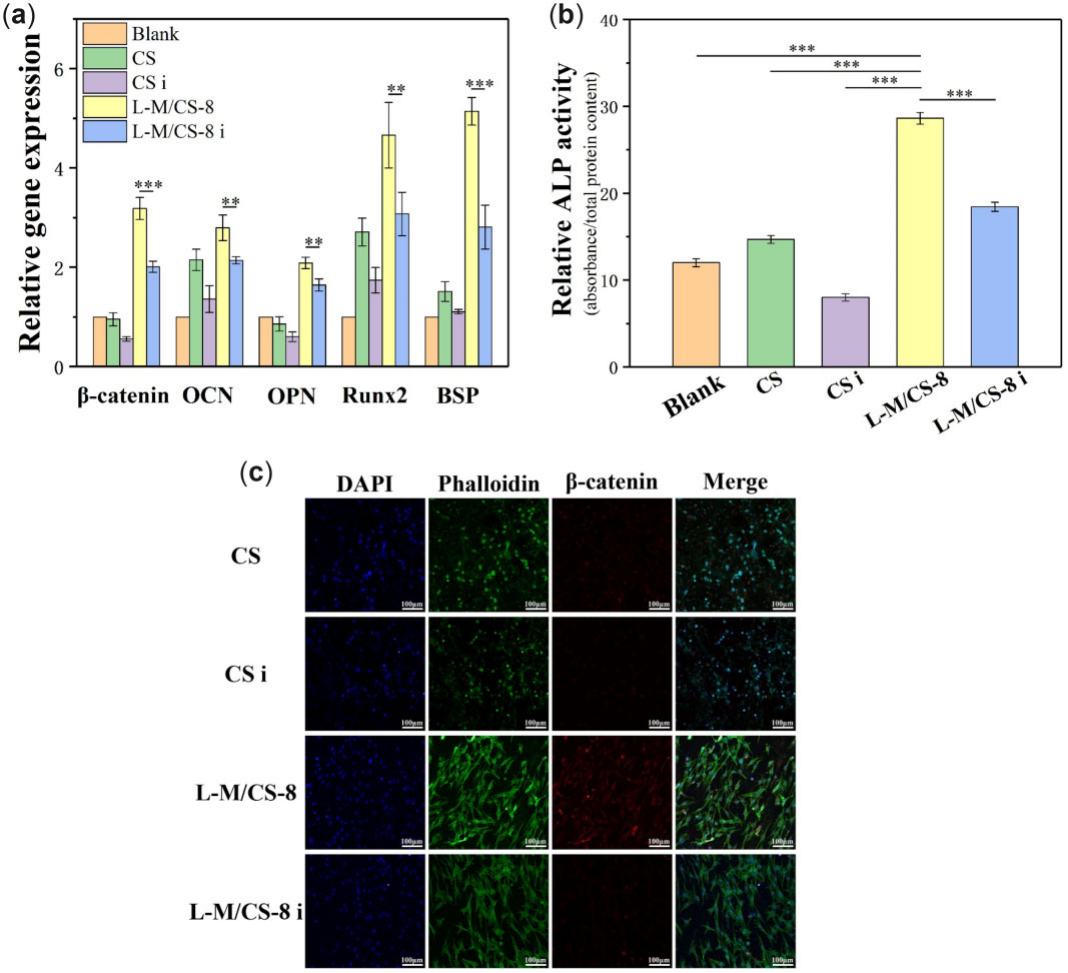
(**a**) Wnt signaling pathway-related genes and osteogenesis-related genes expression of rBMSCs for 7 days (*n* = 4). (**b**) The relative ALP activity cultured for 3 days (*n* = 3). (**c**) Immunofluorescence images (The third column for β-catenin protein expression) of cells cultured for 3 days. ‘i’ means the addition of Wnt signaling pathway inhibitor DDK1 (0.5 μg·ml^−1^). It was showed that the inhibition of Wnt signaling pathway decreased expression of osteogenesis-related genes, indicating that the promotion of osteogenic differentiation by L-M/CS-8 was Wnt signaling pathway-dependent. (*P < 0.05, **P < 0.01, ***P < 0.001)

To confirm the speculation, cells were treated with DKK1 (0.5 μg·ml^−1^). The protein expression, bone-related genes and ALP activity of cells were studied after treating with DKK1. The expression of osteogenesis-related genes decreased apparently after treating with DKK1 for L-M/CS-8 and CS (Fig. 7a). Therefore, the osteogenic differentiation of rBMSCs cultured with L-M/CS-8 or CS reduced significantly once the Wnt signaling pathway was inhibited. The immunofluorescence images revealed the amount of β-catenin protein at a lower level in L-M/CS-8 i (i means the inhibition of Wnt signaling pathway) in compared with L-M/CS-8 (Fig. 7c). ALP activity also exhibited the similar results that ALP activity decreased after treating with DKK1 (Fig. 7b). Above all, the inhibition of Wnt signaling pathway significantly influenced the osteogenic differentiation of rBMSCs cultured with L-M/CS-8 or CS. Cells cultured with L-M/CS-8 exhibited the remarkable osteogenic bioactivity, while the bioactivity was inhibited once the Wnt signaling pathway was inhibited. Therefore, the osteogenic differentiation was Wnt signaling pathway-dependent.

## Conclusion

In our study, inspired by the toughness of natural materials, the laminated calcium silicate-based bioceramics with high toughness were fabricated successfully by the directional assembly-sintering approach. The laminated structure had been shown to promote the mechanical properties through crack deflection and crack branch. In addition, the prepared L-M/CS bioceramics exhibited the more suitable degradability in compared with CaSiO_3_ bioceramics. Moreover, the L-M/CS bioceramics not only promoted the adhesion and proliferation of rBMSCs but also stimulated the osteogenic differentiation. Meanwhile, the ALP activity and the expression of Wnt signaling pathway-related genes of rBMSCs could be apparently enhanced by the L-M/CS bioceramics. The related mechanism of promoting osteogenic differentiation was involved with the Wnt signaling pathway. Above all, the prepared L-M/CS bioceramics with laminated structure exhibited the superior mechanical properties and could promote the osteogenic differentiation of rBMSCs, which made it had great potential for clinical application as bone implants.

## Supplementary data

Supplementary data are available at *REGBIO* online.

## Author contributions

J.H. performed the preparation of materials and characterization the physicochemical properties. D.Z. performed *in vitro* biological experiments. J.X. provided helps in article writing. T.L. provided helps about the preparation of samples. D.R. gave some directions on the sintering of materials. J.H. and C.W. proposed the idea and designed the experiments. C.W revised the manuscript.

## Funding

This work was supported by the National key Research and Development Program of China (2021YFA0715700), the Natural Science Foundation of China (32130062), Shanghai Pilot Program for Basic Research-Chinese Academy of Science, Shanghai Branch (JCYJ-SHFY-2022-003).

*Conflicts of interest* *statement.* None declared.

## Supplementary Material

rbac055_Supplementary_Data
