# Supplementary material for: Bioinspired laminated bioceramics with high toughness for bone tissue engineering
Source: Regen Biomater. 2022 Aug 22;9:rbac055. doi: 10.1093/rb/rbac055 (PMC9438744; doi:10.1093/rb/rbac055)
Supplement: rbac055_Supplementary_Data [file rbac055_supplementary_data.pdf]

# *Supporting Information*

## **Bioinspired Laminated Bioceramics with High Toughness for Bone Tissue Engineering**

Jinzhou Huang[a,b], Dong Zhai[a], Jianmin Xue[a], Tian Li[a], Dudi Ren[a], Chengtie Wu[a,b,*]

[a] State Key Laboratory of High Performance Ceramics and Superfine Microstructure, Shanghai Institute of Ceramics, Chinese Academy of Sciences, 1295 Dingxi Road, Shanghai 200050, P. R. China.

[b] Center of Materials Science and Optoelectronics Engineering, University of Chinese Academy of Sciences, 19A Yuquan Road, Beijing 100049, P. R. China.

* Corresponding author:

Chengtie Wu

E-mail: chengtiewu@mail.sic.ac.cn; Tel: +86-21-52412249.

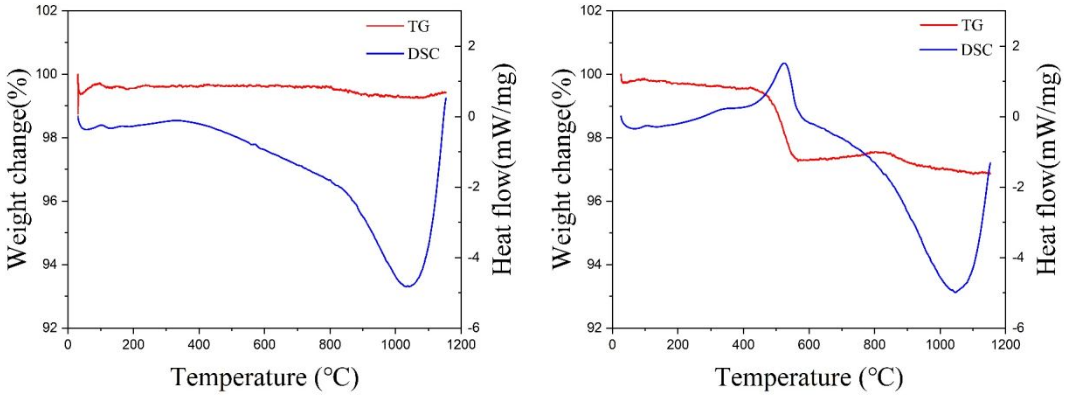


**Figure S1. The thermogravimetric and differential scanning calorimeter analysis of CS (a) and L-M/CS-8 (b).**

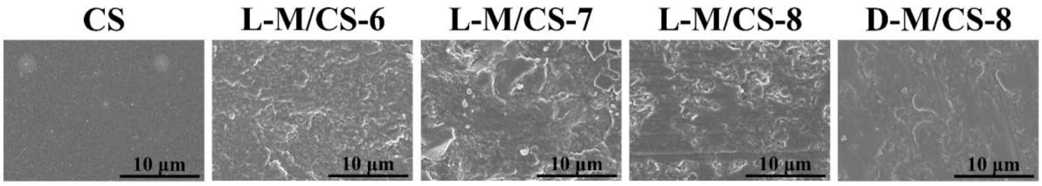


**Figure S2. The SEM images of surface microstructure of CS, L-M/CS-6, L-M/CS-7, L-M/CS-8 and D-M/CS-8 bioceramics.**

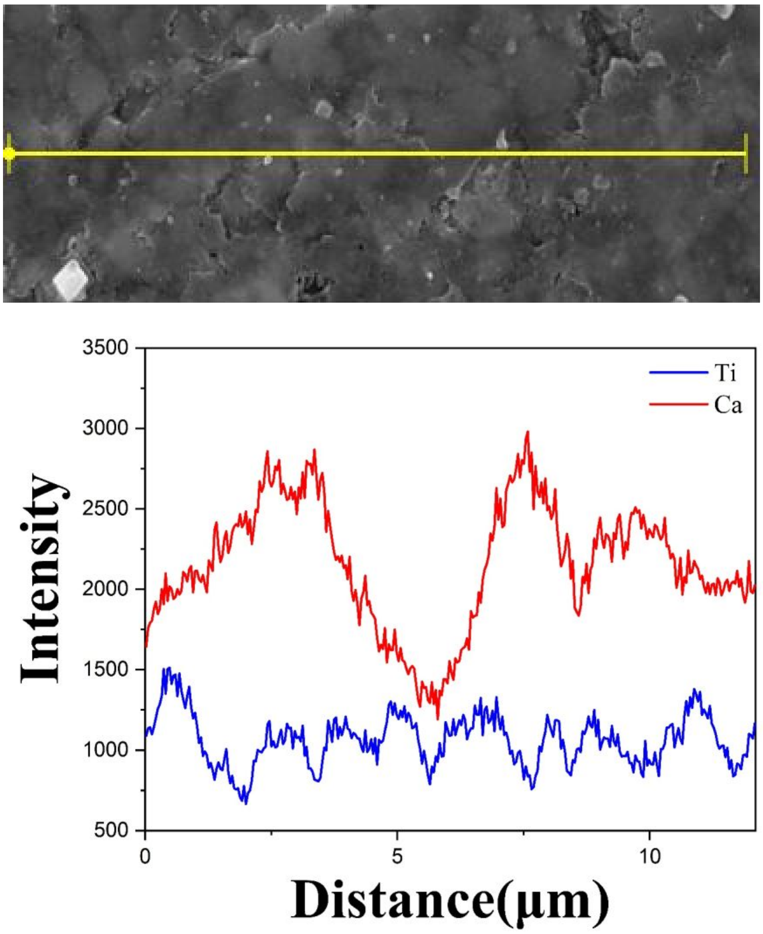


**Figure S3. The element analysis of L-M/CS-8 bioceramic by line scan of SEM. It was observed that Ti element was distributed periodically, which further indicated the existence of the laminated structure.**

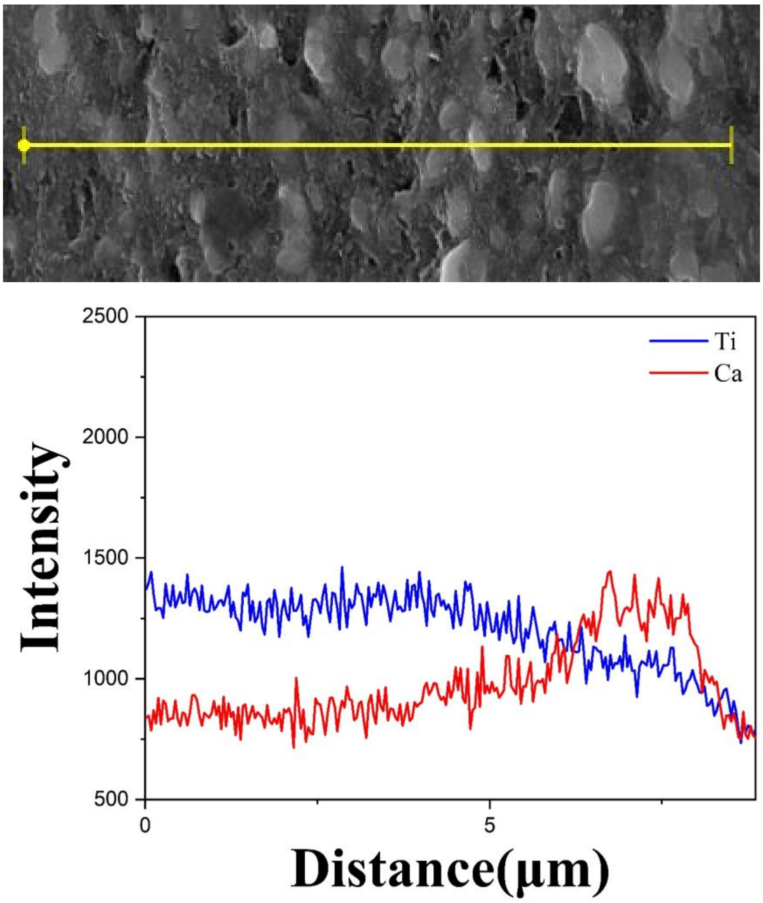


**Figure S4. The element analysis of L-M/CS-7 bioceramic by line scan of SEM.**

**Table S1. The sequences of primers for the genes of Real-time PCR.**

| Gene name | Forward primer sequence (5′→3′) | Reverse primer sequence (5′→3′) |
|---|---|---|
| GAPDH | GAAAGCCTGCCGGTGACTAA | TGGAATTTGCCATGGGTGGA |
| Runx2 | ACTTCCTGTGCTCGGTGCT | GACGGTTATGGTCAAGGTGAA |
| OPN | ATGATGGCCGAGGTGATAGT | ACCATTCAACTCCTCGCTTT |
| OCN | CCGGGAGCAGTGTGAGCTTA | AGGCGGTCTTCAAGCCATACT |
| BSP | GAGCCTCGTGGCGACACTTA | AATTCTGACCCTCGTAGCCTTCATA |
| β-Catenin | TTAAGCCTCTCGGTCTGTGG | GCCGCTTTTCTGTCTGGTTC |
| CK1 | GAGATCCCTTTCCCAGAGTGC | TTTGTGAAGGGCTTCTCGGC |
| APC | AGCAAGTTGAGGCACTGAAGA | TCCCGGCTTCCATAAGAACG |
| Axin | TTATGCTTTGCACTACGTCCCTCCA | CGCAACATGGTCAACCCTCAGAC |
| GSK-3β | GACTAAGGTCTTCCGACCCC | TTAGCATCTGACGCTGCTGT |

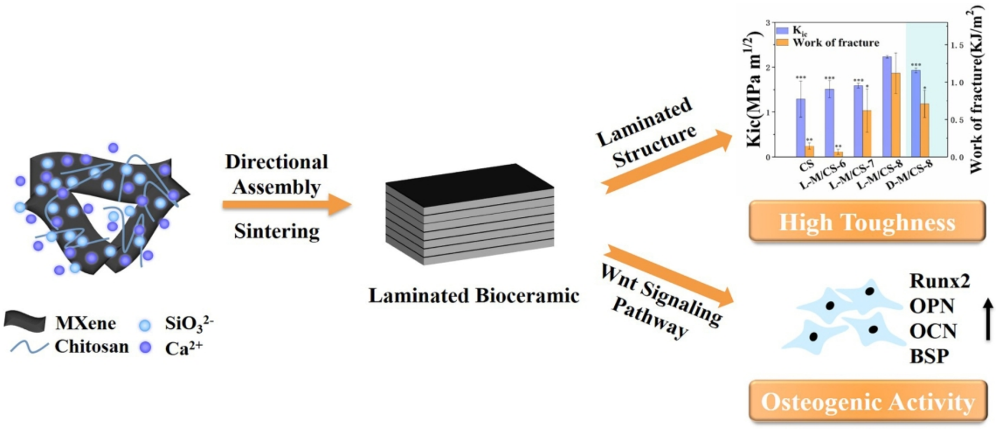


83x35mm (600 x 600 DPI)
